# Stabilization of compressed earth blocks using recycled thermoplastics: experimental study of key parameters

**DOI:** 10.1038/s41598-026-64250-z

**Published:** 2026-08-07

**Authors:** Laila H. Salem, Yassin Aly, Berlant Arab, May Haggag, Safwan Khedr

**Affiliations:** Department of Construction Engineering, The American University in Cairo (AUC), Cairo, Egypt

**Keywords:** Sustainable construction, Compressed earth blocks (CEBs), Recycled thermoplastic stabilization, Polypropylene (PP), Low-carbon materials, Water-efficient construction, Mechanical properties, Thermal conductivity, Engineering, Environmental sciences, Materials science

## Abstract

The construction sector is a major contributor to global resource depletion and environmental pollution, particularly due to its reliance on energy-intensive and water-demanding materials such as cement and fired clay. Moreover, the increasing accumulation of plastic waste and the growing scarcity of freshwater resources present critical sustainability challenges. These intersecting issues highlight the urgent need for innovative, low-carbon, and water-efficient building materials. Among sustainable construction approaches, earth construction offers a viable solution by utilizing natural, locally available materials with minimal environmental impact. This study introduces a cement-free, low-water alternative through the development of recycled thermoplastic-stabilized Compressed Earth Blocks (CEBs) incorporating shredded recycled plastic with heat application. The experimental program comprised 54 mix-designs composed of soil, sand, gravel, lime, water, and plastic, with lime content fixed at 10%. Key variables included plastic content (5%, 10%, 15%), water content (10%, 12.5%, 15%), soil-to-sand ratio (1:1 and 1:1.25), and gravel proportion (5%, 15%, 25%). The blocks were tested for dry compressive strength (DCS), wet compressive strength (WCS) (i.e., following water immersion), and thermal conductivity. The 1:1 soil-to-sand ratio yielded the best performance, achieving a maximum dry strength of 9.93 MPa, wet strength of 4.08 MPa, and minimum thermal conductivity of 0.27 W/m·K. Statistical analysis showed that plastic content had the strongest influence on DCS, while the soil-to–sand ratio governed WCS. Water content was the most influential factor affecting thermal conductivity, whereas the remaining variables showed weak or non-significant effects. The selection framework produced three high-performing mixes (10% lime, 10% water, 10% plastic) with gravel contents of 5%, 15%, and 25%. These mixes advanced through successive performance thresholds, resulting in a refined set of combinations exhibiting strong structural, improved durability, and thermal characteristics. A preliminary economic assessment indicated that the proposed blocks are cost-competitive with conventional concrete bricks. However, this assessment is limited to raw material costs and does not include life-cycle cost or environmental impact analysis. The study avoids cement usage and incorporates recycled plastic waste, although no quantitative assessment of carbon reduction or environmental performance was conducted.

## Introduction

In an attempt to alleviate the impact of climate change, many countries have aligned with the Paris Agreement^1^, a global treaty adopted in 2015 aiming to limit global warming to well below 2 °C, has committed to reducing greenhouse gas (GHG) emissions, targeting a conditional 20% reduction by 2030 across the electricity, oil and gas, and transportation sectors^2^. This commitment is part of the worldwide sustainable development strategy, which includes emission targets, policy reforms, investments, and adaptation measures to support sustainable development by 2030. Achieving this target will require transitioning to cleaner energy sources, improving fuel efficiency, and investing in sustainable infrastructure and technologies^3^. Given that the construction industry is responsible for 38% of global CO₂ emissions^4^, it is key to provide greener alternatives to support the sustainability of such industry. Among the most unsustainable construction materials is Cement which remains one of the most widely used building materials in the world, valued for its strength, availability, and versatility^5^. However, its environmental cost is increasingly unsustainable, with its production alone contributing about 8% of global CO₂ emissions^6^. Another dominant construction material is traditional clay bricks, which are considered affordable and easy to manufacture. However, their manufacturing contributes significantly to the climate crisis, consuming vast amounts of energy. Current models estimate global fired clay brick consumption at 2.18 gigatonnes annually, resulting in approximately 500 million tonnes of CO₂ equivalent emissions, or 1% of global greenhouse gas emissions^7^. Beyond its carbon footprint, construction drives rapid resource depletion, especially in regions already suffering from environmental stress. Concrete production further compounds this issue: in 2012, water withdrawals for the concrete industry accounted for 9% of all industrial withdrawals, representing 1.7% of global freshwater use^8^. This intensifies pressure on water-stressed regions, where increased competition for water threatens agriculture, biodiversity, and human livelihoods.

The last several decades have witnessed the proposal of multiple alternative construction methods noted for their sustainability. Among such promising methods is earth construction^9^, which focuses on utilizing natural, locally sourced materials found on-site, such as soil, sand, and clay, while reducing reliance on industrially produced materials like cement, offering a more environmentally conscious building practice^10^. By relying on renewable and reusable materials and minimizing waste, earth construction also contributes to the circular economy, keeping resources in use for longer and lowering the carbon footprint of buildings. Earth construction can take the form of rammed earth, compressed earth blocks (CEBs), or adobe and has been practiced for over 9,000 years^11^. Archaeological findings show that mud brick (adobe) houses dating back to 8000–6000 BC were discovered in Russian Turkestan^12^, and rammed earth foundations have been traced to around 5000 BC^13^. This long-standing tradition highlights the durability and resilience of earth as a building material, making it a viable modern solution for sustainable construction. Across most hot-arid and temperate regions, earth has historically been the dominant building material, and even today, it is estimated that between 650 and 700 million people still currently inhabit buildings constructed from natural materials such as rammed earth, adobe blocks, wattle and daub, and CEBs^14^.

Traditionally, CEBs are created by compacting a moistened mixture of subsoil and sand, with stabilizers to enhance strength and durability, into dense, uniform blocks that are then air-dried without the need for high-temperature firing. In comparison to fired clay bricks, CEBs are environmentally superior not only due to their lower energy demands but also because they do not require deep soil excavation^15^. From a sustainability perspective, CEBs present a range of environmental and economic benefits: they minimize carbon emissions, reduce water consumption, and are often manufactured on-site using locally available materials, contributing directly to the circular economy by reducing transport emissions and supporting closed-loop resource use^16^. CEBs are also highly adaptable and have been successfully applied across diverse regions, aligning with local construction practices, labor availability, and climatic conditions. In many areas of Africa, Asia, and Latin America, they are increasingly used in housing, educational, and community infrastructure, offering a low-cost, environmentally responsible solution tailored to regional needs^17^.

Stabilization in CEBs is essential to improve their structural performance and long-term durability. Unstabilized CEBs can degrade over time when exposed to moisture or fluctuating weather conditions, leading to structural deterioration^18^. This stabilization can take the form of mechanical, physical, or chemical techniques. Chemical stabilizers like cement and lime are commonly used to enhance CEBs’ strength and durability. Cement increases compressive strength and water resistance but has a high carbon footprint by creating an inert matrix that opposes movement. It was shown that an increase in cement content up to 10.91% combined with 11.40% water resulted in a compressive strength of 15.15 MPa, which exceeded ASTM standards^19^. On the other hand, lime offers a more sustainable option by improving moisture resistance and stability through chemical reactions with water and clay, forming stable chemical bonds between clay crystals through an exothermic hydration reaction^20^. More sustainable stabilizers for CEBs aim to enhance mechanical strength and durability while minimizing environmental impact. Unlike traditional stabilizers such as cement and lime, which are energy-intensive and contribute to carbon dioxide emissions, sustainable alternatives include agricultural by-products like rice husk ash, wheat straw, or natural fibers, and industrial or recycled materials such as fly ash, slag, and waste plastics. These materials not only reduce reliance on virgin resources but also divert waste from landfills, promoting circular economic principles^21^.

Investigations into agricultural-based stabilizers revealed that a mixture of 8% lime with 1.5% rice straw can markedly improve erosion resistance, resisting water soaking for over 45 h^22^. While lime improves workability and may reduce plasticity through mechanisms commonly attributed to cation exchange and carbonation, it cannot provide sufficient strength on its own. Moreover, lime contains calcium hydroxide, which, in the presence of water and pozzolanic materials, siliceous or siliceous-aluminous compounds, is widely understood to undergo a pozzolanic reaction. This reaction is generally considered to produce cementitious compounds, primarily calcium silicate hydrates (C–S–H), which are expected to contribute to the material’s strength and durability, though direct microstructural confirmation was beyond the scope of this study. The integration of lime with pozzolanic additives such as rice husk ash (RHA) has been shown in the literature to promote such cementitious reactions, and consistent with this, a 75% increase in compressive strength reaching 7 MPa and a 60% increase in flexural strength reaching 2.9 MPa were recorded^23^. Evidence from a systematic review of 45 studies indicates that the inclusion of ash-based additives and agricultural by-products, including RHA, improves durability and strength, while natural fibers increase crack resistance, though they may decrease compressive capacity^24^. Experimental results indicate that replacing 10% of cement with RHA increased compressive strength to 6.4 MPa compared to 5.34 MPa for the control samples, while also contributing to a reduced environmental impact^25^. Similarly, substituting 7.5% of clay with RHA was found to increase compressive strength by 25.7% and reduce water absorption^26^. Other research highlights that incorporating 3% achronal and 1.5% rice straw significantly enhances compressive strength and durability^18^.

Further investigations into natural fiber reinforcement strategies highlight the potential of lignocellulosic additives in enhancing the mechanical and durability performance of CEBs. The incorporation of areca fibers in proportions ranging from 0 to 3% by dry soil mass demonstrated substantial improvements in strength characteristics, with compressive strength increasing by up to 436.38%, split tensile strength by 358.08%, and flexural strength by 82.47%^27^. Durability assessments further revealed enhancements in wet compressive strength (up to 100.42%) and controlled water absorption, with an optimal fiber content identified at 2%^27^. Similarly, the inclusion of Vetiver straw fibers (Chrysopogon zizanioides) has been shown to improve ductility and reduce volumetric shrinkage and bulk density, although gains in compressive strength remained marginal over time^28^. Complementary findings indicate that Vetiver shoot reinforcement can yield up to a 38% increase in compressive strength and a 202% improvement in ductility index, underscoring its effectiveness in enhancing deformation capacity and crack resistance^29^. In parallel, the development of fiber-reinforced geopolymer earth bricks incorporating industrial by-products such as fly ash and ground granulated blast-furnace slag, combined with 1% coir fiber, achieved compressive and flexural strengths of 5.93 MPa and 3.43 MPa, respectively, alongside a 40% increase in compressive strength and improved resistance to acidic and sulfate environments^30^. Collectively, these findings emphasize that while natural fibers primarily enhance ductility, toughness, and durability, their integration with cementitious or geopolymeric binders is essential to achieving balanced improvements in strength and long-term performance. To position the present findings within the broader body of recent CEB stabilization research, Table 1**.** summarizes six recent studies employing alternative stabilizer systems, including industrial by-products, recycled binders, and waste-derived aggregates. The comparison spans stabilizer type, dry compressive strength, durability indicators, thermal conductivity, and cost or sustainability considerations, providing a clearer basis for evaluating the relative performance of the proposed approach.

**Table 1:** Comparative performance of recent CEB stabilization studies.

Stabilizer type	Dry compressive strength (MPa)	Durability indicator	Thermal conductivity (W/m·K)	Cost	Sustainability notes
Acrea fibers^27^	Up to 2.28	WCS up to 1.60 MPa Water Absorption as low as 12.54%	Not reported	Not reported	Valorizes agricultural waste
Vetiver straw fibers^28^	Up to 1.36	Water Absorption as low as 15.41%	Not reported	Not reported	Valorizes agricultural waste
Fly Ash, sewage sludge & cement^31^	Up to 11.36	WCS up to 9.43 MPa Water Absorption as low as 13.9%	Not reported	Not reported	Offsets carbon savings
Recycled cement (RC)^32^	Up to 10.9	Coefficient of abrasion 43.6	Not reported	Not reported	Sources RC from waste concrete; higher water demand than OPC
Blast furnace slag, cement & lime^33^	Up to 14.31	Not reported	Not reported	Not reported	Reduces industrial by-product (BFS)
Sewage sludge ash & cement^34^	Up to 5.42	WCS up to 6.39 MPa Water absorption as low as 10.16%	Not reported	Not reported	Diverts heavy-metal-bearing SSA from landfill
Ceramic waste powder (CWP) ^35^	Up to 8.96	WCS up to 5 MPa Water absorption as low as 11%	Not reported	≈11% cost reduction from cement	Eliminates kiln firing during CEB production
Crushed brick waste, cement & lime^36^	Up to 9.57	WCS up to 8.43 MPa Water absorption as low as 10.62% No sign of efflorescence was observed	Not reported	Not reported	Valorizes construction & demolition waste

Alternatively, the world faces challenges from the unsustainable production and disposal of plastic. Approximately 430 million tons of plastic are produced annually, with two-thirds becoming short-lived products and waste. Furthermore, plastic production, largely derived from fossil fuels, was responsible for 3.4% of global greenhouse gas emissions as of 2019^37^. As such, various types of thermoplastics were considered for soil stabilization, including polyethylene (PE), polypropylene (PP), polystyrene (PS), and PET. Each type exhibits distinct melting points, degradation behavior, and mechanical characteristics that may influence their performance when used as stabilizers^38^. Importantly, PP is recognized as one of the safest plastics for human contact, particularly in food packaging and medical applications. It does not contain bisphenol A (BPA) or phthalate plasticizers, chemicals commonly associated with hormonal disruptions in humans. Also, it is considered generally inert and thermally stable up to approximately 130–170 °C^39^. Incorporating 1% polyethylene terephthalate (PET) into CEBs was shown to enhance compressive strength and reduce block weight, though increasing the plastic content up to 7% led to diminished erosion resistance. Although thermoplastics do not chemically react with soil particles, they are thought to contribute to strength enhancement by forming a cohesive matrix that physically binds particles, a mechanism that appears particularly effective when used in combination with lime, which is associated with densification and microstructural improvement. While these interactions are plausible based on observed macroscopic behavior, direct microstructural confirmation was not provided in that study. The research in question demonstrated improved strength, though compressive strengths reached only around 1 MPa, with higher strength and enhanced durability identified as targets for further investigation^40^.

In light of these environmental and resource challenges, this study proposes a cement-free, low-water alternative for producing construction blocks by developing thermoplastic-stabilized CEBs. The research addresses the dual crises of construction-related emissions and plastic waste accumulation, offering a solution that aligns with the sustainable development goals. As such, this work uses locally sourced soil, sand, gravel, lime, and recycled plastic as stabilizers to produce high-strength, water-efficient building blocks. The methodology involved testing various thermoplastic-stabilized CEB specimens with heat application to enhance bonding and performance. The experimental phase covered 54 systematically varied mix combinations, with lime content fixed at 10%. Each mix produced six replicate samples, totaling 324 blocks. These were grouped by plastic content (5%, 10%, and 15%) while varying water content (10%, 12.5%, 15%), soil-to-sand ratio (1:1, 1:1.25), and gravel percentage (5%, 15%, 25%). The blocks were tested for dry compressive strength (DCS), wet compressive strength (WCS) after water immersion, and thermal conductivity to evaluate their mechanical, water durability, and thermal performance. In addition, a preliminary cost assessment was undertaken to evaluate the economic feasibility of the proposed blocks. The primary aim is to develop mix design that meets or exceeds structural performance benchmarks, reduces reliance on cement, and demonstrates a scalable, circular construction approach suitable for resource-scarce, climate-vulnerable regions. The study focuses on material development and performance evaluation, while environmental and economic sustainability claims are restricted to qualitative discussion due to the absence of life-cycle assessment data. By demonstrating a resource-efficient approach to producing earth blocks, the work contributes to a practical model suitable for areas facing material and economic constraints.

## Experimental investigation

### Classification of testing specimens

The experimental program was structured in two main phases: initial testing and parametric testing. The initial phase focused on validating the feasibility of incorporating shredded recycled plastic with heat application into CEBs. Six specimens were prepared using a uniform mix of Sand and Gravel (55%), Silt/Clay (45%), Lime (10%), Water (11%), and 10% shredded thermoplastic as an additional stabilizer. These proportions were selected in line with Egyptian Earth Construction Code of Practice. A control mix without thermoplastic was also produced to enable direct comparison. The results showed a substantial performance enhancement due to thermoplastic incorporation: dry compressive strength increased from 2.55 MPa in the control to 9.70 MPa, wet compressive strength improved from 0 to 2.00 MPa while thermal conductivity decreased from 1.43 W/m·K to 1.05 W/m·K, indicating improved insulation^41^.

After validation, the study advanced to the parametric testing phase, which investigated how variations in mix proportions affect the performance of thermoplastic-stabilized CEBs. This stage employed a full factorial design to generate 54 unique mix combinations by varying four key parameters: soil-to-sand ratio, plastic content, water content, and gravel content. The lime content was fixed at 10%, as preliminary studies indicated no significant improvement in performance beyond this percentage^24,42^. The selected water contents (10%, 12.5%, and 15%) were determined based on both Paul et al. values and preliminary verification tests^43^. Previous studies on similar clayey soils indicate that the plasticity index typically ranges from 10–20%, corresponding to soils with medium to low plasticity. These ranges were therefore used as a reference to select a representative moisture content interval for the present study. To experimentally verify the appropriate water content, the ball drop test recommended by the Egyptian Code of Practice was performed. In this test, a ball was formed from the soil mixture in the palm of the hand and dropped from a height of approximately 1 m. If the ball breaks into three to four parts, the moisture content is considered suitable for compaction. The preliminary results indicated that a water content of approximately 11% provided the most suitable workability and cohesion for the mixture. However, in order to investigate the influence of moisture variation on the mechanical performance of the specimens, a wider range of water contents (10–15%) was adopted in the experimental program. This approach is consistent with previous studies investigating soil stabilization mixtures within the typical optimum moisture content range reported by Paul et al.^43^.

The selected plastic contents (5%, 10%, and 15%), on the other hand, were determined based on a combination of factors, including existing literature, preliminary experimental results, and the opportunity cost associated with plastic material usage. Previous research by Isaac et.al on the production of compressed earth bricks (CEBs) incorporating soil and varying proportions (0%, 1%, 3%, and 7%) of shredded waste plastic demonstrated that an optimal plastic content of 1% (by weight), with particle sizes smaller than 6.3 mm, resulted in a 244.4% increase in compressive strength^40^. In addition to plastic, alternative stabilizing materials were also considered. Notably, studies utilizing shredded waste rubber tire chips as reinforcement examined proportions of 5%, 10%, and 15%, providing a comparative basis for selecting similar percentage ranges in this study^44^. During the preliminary testing phase, samples containing 10% plastic exhibited a compressive strength of 9.7 MPa, corresponding to an improvement of approximately 280% compared to the control sample. However, further analysis indicated that 10% plastic content would significantly increase the production cost of the bricks. Consequently, lower plastic percentages were selected for further investigation to assess whether comparable mechanical performance could be achieved while reducing overall material costs.

Additionally, the selected percentage contents of gravel (5%, 15%, and 25%) were determined based on guidance from the Egyptian Code of Practice for Earth Construction, which recommends an optimal gravel content of approximately 15% for soil stabilization and improved mechanical performance. This value was adopted as a benchmark to ensure compliance with established local engineering standards and to provide a reliable reference point for material behavior. To further investigate the influence of aggregate content on the performance of the CEBs, lower and higher percentages (5% and 25%) were also incorporated into the experimental program. The specimens were classified in a hierarchical manner: first by soil-to-sand ratio (S/S) into two main groups (1:1 and 1:1.25), then by plastic content (5%, 10%, and 15%), followed by water content (10%, 12.5%, and 15%), and finally by gravel content (5%, 15%, and 25%). For example, (Specimen 1) has a soil-to-sand ratio of 1:1, with 5% plastic, 10% water, 5% gravel, and a constant lime content of 10%. Table 2 presents the classification framework used in this study.

**Table 2:** Hierarchical classification of specimens.

Soil-to-sand ratio (S/S)	Plastic content (%)	Water content (%)	Gravel content (%)	Number of specimens
(1:1) (1:1.25)	5	10	5, 15, 25	18
		12.5	5, 15, 25	
		15	5, 15, 25	
	10	10	5, 15, 25	18
		12.5	5, 15, 25	
		15	5, 15, 25	
	15	10	5, 15, 25	18
		12.5	5, 15, 25	
		15	5, 15, 25	

This hierarchical classification resulted in 27 distinct mixes within each soil-to-sand category, yielding a total of 54 unique mixes across all groups. For each mix configuration, six replicate specimens were produced: three for determining the average dry compressive strength (destructive), two for measuring thermal conductivity (non-destructive), and three for evaluating the effect of 24-h water immersion on compressive strength (destructive). In total, 324 specimens were tested to assess the mechanical strength, water durability, and thermal performance of the CEB blocks.

### Materials and characterization methods

Soil, the base raw material for CEBs, acts as the main binder in which the clay and silt fractions enhance cohesion and moldability. In this study, locally sourced soil was collected from a construction site in Ramsis, Cairo, Egypt. Representative samples were obtained from depths of 0.5 m or more, ensuring that the soil used was structurally stable and free from organic matter. The soil was air-dried and sieved on sieve No. 4 (4.75 mm) to remove oversized aggregates and any clay clumps. Where necessary, the soil was mechanically broken down to achieve uniform gradation. Mechanical and physical properties were determined through sieve and hydrometer analyses (ASTM C136/C136M-19; ASTM D7928), Atterberg limits (ASTM D4318), and specific gravity tests (ASTM D854). The Unified Soil Classification System (USCS) was applied to confirm suitability for CEB production. In addition to the soil, well-graded natural sand sourced from New Cairo, Egypt was incorporated to enhance the performance of the CEBs by reducing shrinkage, improving strength, and enhancing workability. The sand used passed through a No. 4 (4.75 mm) sieve, ensuring compatibility with the soil matrix and contributing to optimal particle packing and densification. The sand was tested for specific gravity in accordance with ASTM D854, and its particle size distribution was determined using sieve analysis as per ASTM C136/C136M-19. Along with sand, locally sourced crushed natural gravel from the New Cairo area in Egypt, was incorporated as a coarse granular material to enhance compressive strength and reduce shrinkage. Gravel retained on the 4.75 mm (No. 4) sieve and passing the 9.5 mm (No. 3½) sieve was used, ensuring proper interlocking between particles while avoiding large voids and weak zones in the block structure. The particle size distribution of the gravel was determined using sieve analysis in accordance with ASTM C136/C136M-19.

To further improve stabilization, lime was utilized to stabilize the soil when combined with water, and to enhance the strength and durability of the CEB specimens. In the presence of water, lime is understood to promote the formation of calcium silicate hydrates, which are believed to create stable bonds between clay and sand particles, potentially contributing to improved long-term performance. Hydrated lime was sourced from the El Minya region, supplied in 25 kg bags, and used at a constant dosage of 10% by weight of the dry mix in all specimens. The chemical and physical properties of the hydraulic lime are presented in Table 3**.** Water was added to initiate the hydration of lime and enhance workability. Its amount varies across the parametric mixes as proper moisture content was crucial for lime activation and for aiding the melting and bonding of plastic.

**Table 3:** Chemical and physical properties of the hydraulic lime.

Property type	Property	Value
Chemical	Ca (OH)₂	80–92%
	SiO₂	≤ 0.05%
	MgO	≤ 0.08%
	Fe₂O₃	≤ 0.01%
	Insoluble Residue (O.I)	1.5–4%
Physical	Density	0.60–0.80 g/mL
	Mohs Hardness	2–3
	Whiteness	90%
	Particle Size (fine product)	D90 = 90 µm, D50 = 20.45 µm

**Fig. 1:**
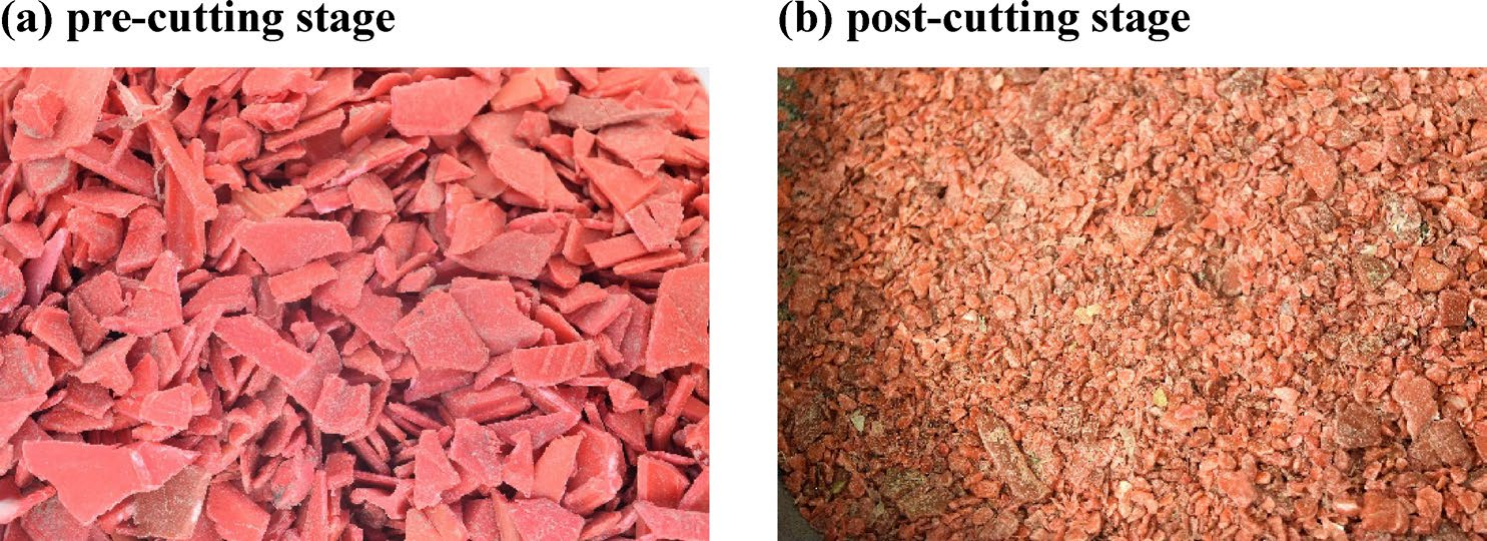
Plastic waste: (**a**) pre-cutting stage, (**b**) post-cutting stage.

In addition to soil, sand, gravel, lime, and water, recycled PP was selected for this study due to its relatively low cost, favorable melting behavior, and widespread availability, primarily sourced from post-consumer plastic packaging. The characteristic properties of the recycled PP used in this study are presented in Table 4. PP waste was collected from waste transit stations in Cairo, Egypt, in the pre-cutting stage (Fig. 1a), then thoroughly washed and cut to the required sizes for experimental purposes in the post-cutting stage (Fig. 1b). The material was subsequently crushed to 2–3 mm and shredded into small pieces passing a No. 4 (4.75 mm) sieve. During block curing, the plastic was heated only to its melting point (approximately 155 °C), thereby avoiding combustion and preventing CO₂ emissions. The molten plastic acted as a binder, creating strong interparticle bonds, minimizing internal voids, and reducing the risk of cracking.

**Table 4:** Characteristic properties of recycled PP.

Property/characteristic	Description/value
Material type	Recycled PP
Source	Post-consumer plastic packaging collected from waste transit stations in Cairo, Egypt
Pre-processing stage	Collected in pre-cutting stage; washed and cut in post-cutting stage
Particle size	Crushed to 2–3 mm; shredded to pass No. 4 (4.75 mm) sieve
Density (g/cm^3^)	0.897–0.900
Melting point (°C)	~ 155 °C (heated only to melting point during block curing)
Tensile strength (MPa)	16.1–24
Flexural modulus (MPa)	700–850
Young’s modulus (MPa)	700–850
Melt flow index (g/10 min)	0.5–2.14

### Specimen production

**Fig. 2:**
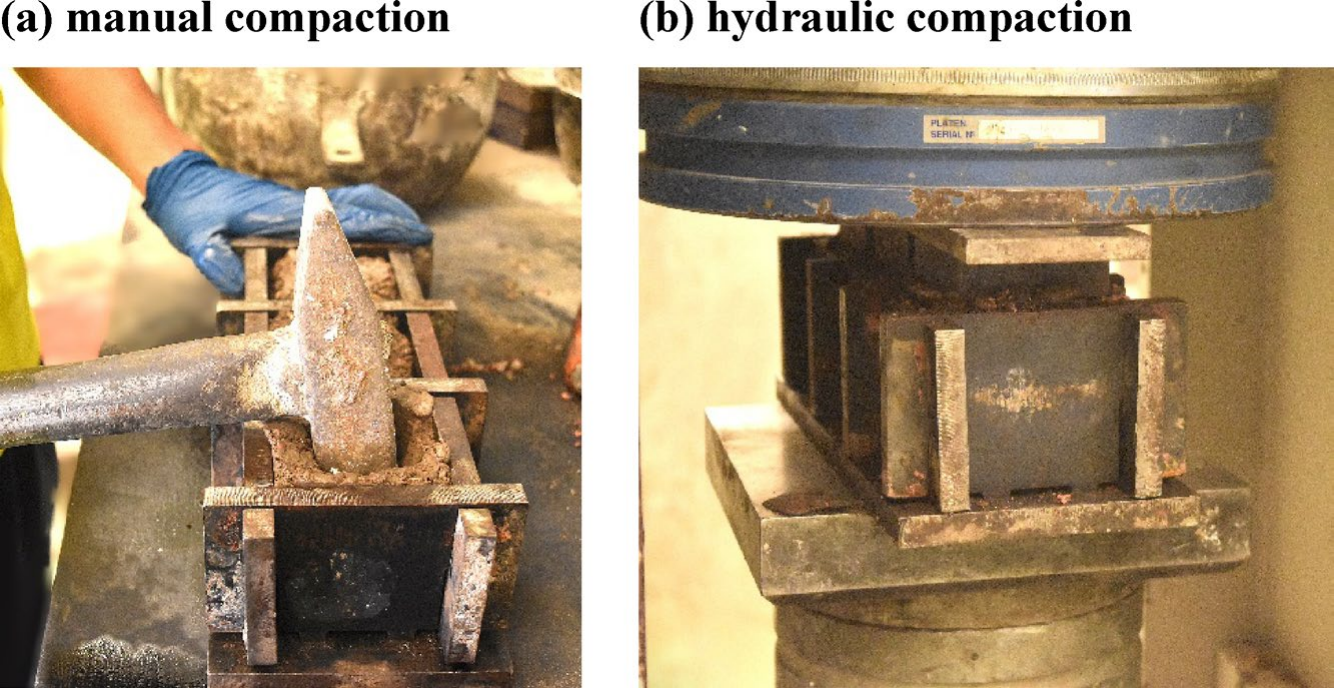
Illustration of the compaction processes: (**a**) manual compaction, (**b**) hydraulic compaction.

For all specimens, the following procedure was applied in batches, with each batch prepared for six specimens having similar content. After preparing all materials according to the specified requirements, all dry materials, including soil, sand, gravel, lime, and recycled shredded plastic, were blended in a rotating pan mixer for approximately 5–10 min. Water was then gradually added during mixing to ensure a homogeneous composition with uniform moisture distribution. The prepared mixture was placed into a metal mold accommodating three cubic specimens, each measuring 7 × 7 × 7 cm, and the mold was lubricated with oil to facilitate demolding. The mixture was introduced in three layers, where each layer was lightly tamped manually to ensure proper distribution and levelling of the material within the mold. Following this preliminary tamping step, the specimens were compacted using a static hydraulic press with a metal plate at a constant pressure ranging between 9 and 12 MPa for 20–30 s, ensuring uniform compaction across all samples. Compaction was controlled by applied pressure rather than impact energy. This method was adopted to provide consistent compaction conditions. It should be noted that the manual tamping was only intended as a preparation step to distribute the mixture within the mold, while the primary compaction energy was applied through the hydraulic press. Final specimen density was not monitored, as each mix possessed a different composition and corresponding density. Figure 2a and b illustrate the manual and hydraulic compaction processes, respectively.

**Fig. 3:**
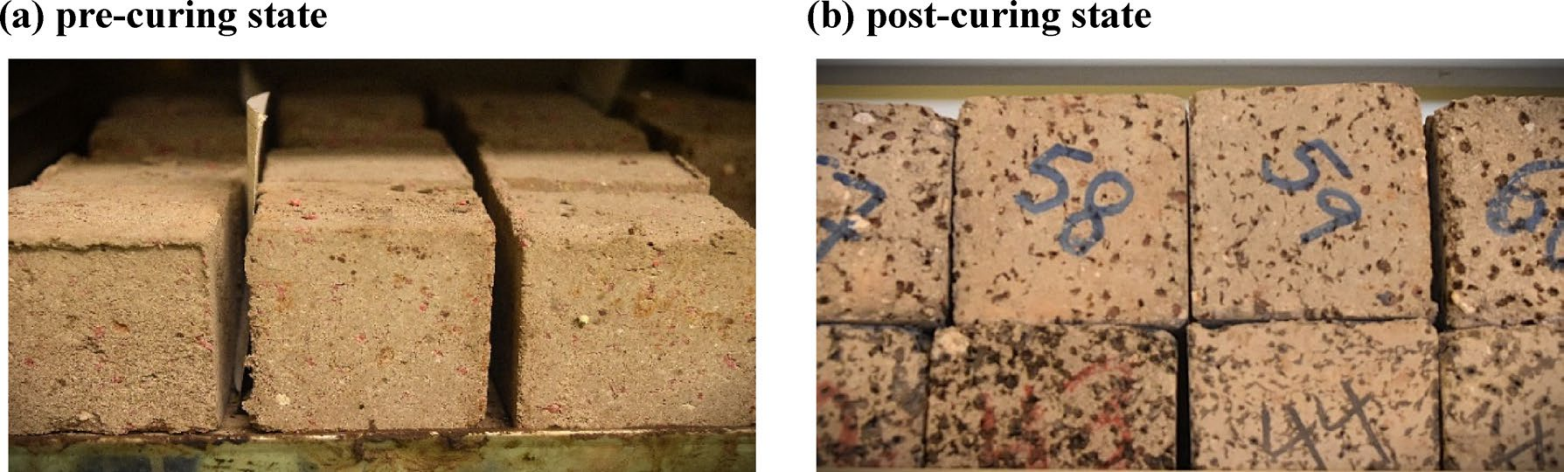
Representative specimens: (**a**) pre-curing state, (**b**) post-curing state.

The compacted specimens underwent initial curing in an oven at 100 °C for 2–3 h to promote early strength gain through lime–water reactions and to prevent specimen damage. After cooling, to prevent cracking, the specimens were demolded and placed in an oven, where the temperature was gradually increased to approximately 155 °C for 3–4 h, enabling the plastic to melt, thereby internally binding the particles without combustion. Representative specimens were observed in both the pre-curing state and after the completion of the curing process (Fig. 3a, b).

### Specimen testing

The specimens were tested for dry compressive strength, wet compressive strength after water immersion, and thermal conductivity to evaluate their performance. Standard procedures were applied for preparation, setup, and measurement, with calculations carried out using the relevant equations to ensure consistency and reliability.

**Fig. 4:**
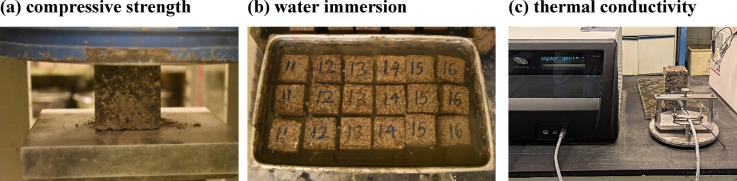
CEB specimen testing: (**a**) compressive strength, (**b**) water immersion, (**c**) thermal conductivity.

**Dry compressive strength:** This test was performed in accordance with ASTM D1633-17. Three specimens were tested after 24 h of cooling using the ELE compression testing machine, which is widely used for this purpose (Fig. 4a). The machine applied the load at a constant rate of deformation without shock to achieve the prescribed strain rate. Alternatively, the load could be applied at a constant rate of stress within the allowable range. The load was applied gradually until it began to decrease steadily, indicating specimen failure. The compressive strength ${f}_{cu}$ was determined using Eq. (1).

1 $${f}_{cu}=\frac{{P}_{max}}{A}$$

where ${P}_{max}$ is the maximum load at failure in Newtons (N), and *A* is the cross-sectional area of the specimen in square millimeters (mm^2^). The reported value represents the average of three specimens for each mix design in megapascals (MPa).

**Wet compressive strength**: The effect of water immersion on compressive strength was evaluated in accordance with the approach outlined in ASTM C140/C140M-24, as moisture resistance is an indicator of the durability and service performance of thermoplastic-stabilized CEB blocks. The test was conducted on an average of three specimens, which were fully submerged in water for 24 h (Fig. 4b) prior to testing. The compressive strength after immersion was then determined using the same procedure and equation as in Eq. (1). This allowed for direct comparison between the compressive strength of dry and water-immersed specimens, thereby quantifying the impact of moisture exposure on mechanical performance. Durability in this study was evaluated using a 24-h water immersion test, consistent with commonly adopted procedures for masonry unit absorption testing. The selected immersion duration follows the methodology described in ASTM C140/C140M-24, which specifies procedures for evaluating the physical properties of masonry units. The 24-h period is used to simulate short-term saturation conditions, such as heavy rainfall or temporary flooding, and indicates the material’s resistance to water ingress. This research was conducted in Egypt, where construction using compressed earth materials is generally intended for relatively dry climatic conditions, and blocks are typically protected with surface finishes or roof overhangs to limit prolonged water exposure. Only the wet compressive strength was evaluated after the 24-h immersion, and the results showed that the proposed mixtures retained a substantial portion of their dry strength, indicating acceptable short-term resistance to water exposure. Nevertheless, the durability assessment in this study is limited to short-term water immersion, and long-term performance may be influenced by repeated wetting–drying cycles and other environmental exposure conditions. Future work should therefore include cyclic wetting–drying tests, as well as extended durability and environmental exposure studies, to provide a more comprehensive evaluation of the proposed blocks.

**Thermal conductivity**: This test was performed in accordance with ISO 22,007–2. Thermal conductivity tests were conducted between two blocks of the same mixture to determine the energy required to change one Kelvin in one meter of the material, expressed in W/m·K. The thermal conductivity affects the thermal comfort of the users and the operational energy demand within the structure; therefore, lower thermal conductivity is desired for sustainable construction. The measurements were conducted using a Transient Plane Source (TPS) apparatus (Hot Disk TPS 5599, sensor radius α = 6.403 mm) (Fig. 4c), with the flat double-spiral sensor sandwiched between two flat faces of the specimen. The sensor acts simultaneously as a heat source and a temperature recorder. A controlled electrical current of 100 mW was applied to the sensor for 20 s, and the resulting temperature rise was recorded as a function of time. Thermal conductivity and thermal diffusivity were calculated from the transient temperature response using Eq. (2) and Eq. (3) ^45^.

2 $$\Delta \overline{T }\left(\tau \right)={P}_{o} /{ (\pi }^{3/2}\cdot \alpha \cdot \mathrm{K})\cdot \mathrm{D}(\uptau )$$

3 $$\tau = \sqrt {t} /\alpha$$

where ΔT̅(τ) is the mean temperature increase of the sensor (K); P_o_ is the total output power of the sensor (W); α is the radius of the TPS sensor (m); K is the thermal conductivity (W·m^−1^·K^−1^); D(τ) is a dimensionless time-dependent function describing the geometry of the sensor; τ is the dimensionless time parameter; t is the measurement time (s); and κ is the thermal diffusivity (m^2^·s^−1^). The TPS method was selected for its rapid, non-destructive measurements, minimal sample preparation, and ability to simultaneously determine multiple thermal properties from a single short measurement, making it well suited for solid block specimens such as compressed earth blocks. The method is formally standardized under ISO 22,007–2 and further validated under ASTM E3088-25 for a broad range of isotropic and anisotropic solid materials, confirming its accuracy and wide applicability.

Regarding the experimental setup, no physical insulation was used at the sample boundaries to minimize edge losses. The TPS method inherently avoids edge effects by ensuring the thermal pulse does not reach the sample boundaries during the measurement period. The measurement time of 20 s was specifically selected to satisfy the infinite medium condition across all tested specimens. Given the variation in thermal diffusivity across all the specimens, the calculated probing depths ranged from 6.80 mm to 15.57 mm, the probing depth remained significantly smaller than the shortest distance from the sensor to any specimen edge, which was 35 mm for the 70 × 70 × 70 mm specimens, yielding a minimum safety margin of 2.25 times the maximum probing depth. Consequently, the measurements remained independent of boundary conditions and edge effects across all specimens, ensuring high data integrity without the need for external insulation. The heating pulse parameters used in the thermal conductivity tests are listed in Table 5**.**

**Table 5:** Heating pulse characteristics used in the thermal conductivity tests.

Parameter	Value
Disk type	Kapton 5501 F2
Sensor radius	6.403 mm
Output power	100 mW
Measurement time	20 s
Initial sample temperature	21 °C
Disk resistance (Rs)	6.75 Ω
Temperature coefficient of resistance (TCR)	0.005059 K⁻^1^

The measured thermal response was dominated by conduction, with negligible influence from convection. The TPS method employed a short heating pulse (20 s) and low power (100 mW), ensuring heat transfer remained confined to the solid matrix. The calculated probe depth (6.80 mm to 15.57 mm) was well within the specimen boundaries, preventing air movement or edge effects. Additionally, the absence of temperature drift during the test confirms that convective mechanisms were not activated, consistent with standard TPS methodology^46^.

Measurements were conducted in a climate-controlled laboratory at a constant ambient temperature of 23 ± 1ºC. To mitigate the influence of ambient fluctuations, specimens were equilibrated in the test environment 24 h before measurement to ensure a uniform initial temperature distribution. Additional thermal insulation was not required during the procedure, as the transient measurement period was sufficiently short (20 s) to prevent convective heat exchange at the specimen boundaries from interfering with the localized heat pulse at the sensor-material interface. This ensures that the recorded temperature rise was strictly a function of the internal heat source and the material’s intrinsic thermal properties. It is acknowledged as a limitation of this study that the cooling phase was not independently analyzed; however, this is inherent to the TPS method rather than a study-specific constraint, as the theoretical model under ISO 22,007–2 is formulated exclusively for the heating transient.

## Results and discussions

### Sand and soil properties and classification

**Fig. 5:**
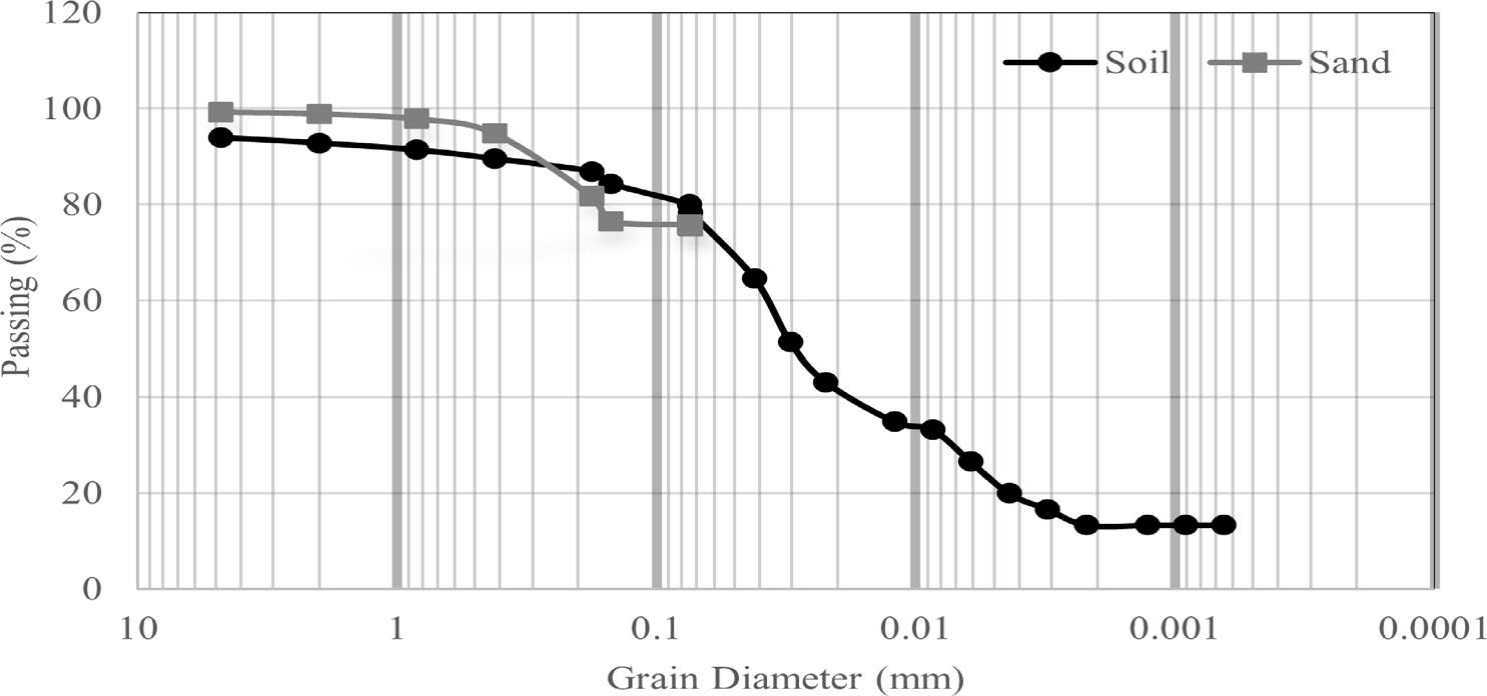
The particle size distribution of the sampled sand and soil for specimens’ preparation.

The materials used in this study were characterized through a series of standard laboratory tests to determine their physical and classification properties. A sieve analysis was first conducted on both sand and soil samples in accordance with ASTM C136/C136M-19, along with a hydrometer analysis following ASTM D7928. The sieve analysis results indicated that more than 50% of both sand and soil samples passed through the No. 200 sieve (Fig. 5), indicating a high fines content, which serves as an initial indicator in the Unified Soil Classification System (USCS).

The specific gravity, determined according to ASTM D854, was found to be 2.57 for sand and 2.78 for soil, and these values were used in the mass calculations for mix design preparation. The liquid limit for soil was obtained following ASTM D4318 and was found to be 32%, while the plastic limit test could not be completed due to the sample’s very low plasticity. Based on these results and the USCS classification, the soil sample was identified as ML- Inorganic Silt with Low Plasticity. A summary of the key physical properties of the tested soil is presented in Table 6.

**Table 6:** Physical properties of the soil.

Properties	Value
Specific gravity	2.78
Liquid limit	32%
Plastic limit	0
Plasticity index	0
% passing sieve No. 200	82%
USCS soil classification	ML- Inorganic Silt with Low Plasticity

### Dry compressive strength

**Fig. 6:**
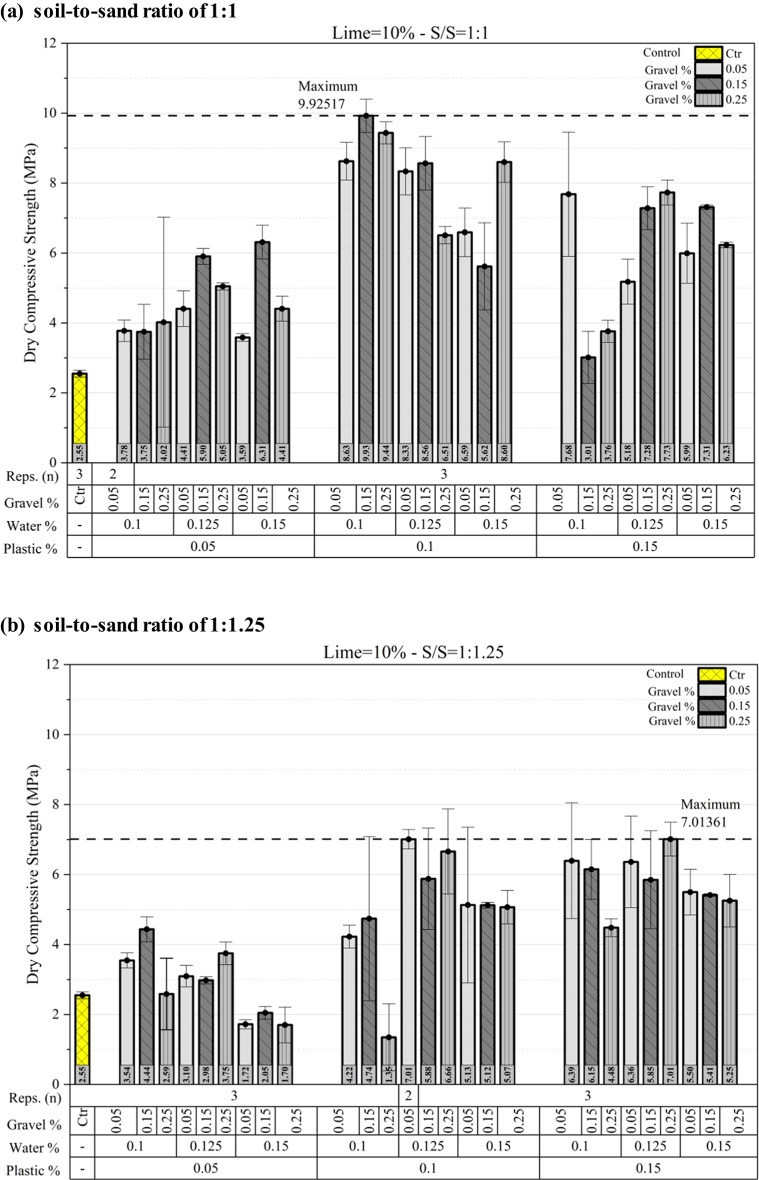
Dry Compressive strength of thermoplastic-stabilized specimens: (**a**) soil-to-sand ratio of 1:1, (**b**) soil-to-sand ratio of 1:1.25. (*Note*: Error bars show the average upper and lower deviation from the mean.)

Figure 6a and b present the variation in DCS of thermoplastic-stabilized specimens with a fixed lime content of 10% at two different soil-to-sand ratios: 1:1 and 1:1.25, respectively. Each bar represents the mean DCS of three replicate specimens (n = 3); for a limited number of mixes, specimen loss during demolding or testing reduced the available replicates to two (n = 2) as marked in the number of replicates row (Reps.). The results demonstrate that DCS is highly sensitive to the combined influence of gravel, water, and plastic contents, with noticeable differences between the two mix ratios. Moreover, the relationship between these parameters and compressive strength is nonlinear, indicating complex interactions between particle packing, water content, and thermoplastic bonding within the matrix. Most thermoplastic-stabilized mixes showed strength levels above the control strength of 2.55 MPa, with several achieving more than three times this value, clearly demonstrating the substantial strength gains achieved through thermoplastic incorporation.

For the 1:1 soil-to-sand ratio (Fig. 6a), the maximum DCS of 9.93 MPa was achieved at 15% gravel, 10% water, and 10% plastic, representing the optimum mix that provides the best balance between particle packing, cohesion, and binder reactivity. Moderate water and plastic contents were found to enhance compaction and matrix bonding, leading to superior strength performance. The optimum conditions for each plastic level were 6.31 MPa at 5% plastic (15% water, 15% gravel), 9.93 MPa at 10% plastic (10% water, 15% gravel), and 7.73 MPa at 15% plastic (12.5% water, 25% gravel). For the 1:1.25 soil-to-sand ratio (Fig. 6b), the maximum DCS recorded was 7.01 MPa at 25% gravel, 12.5% water, and 15% plastic. The results indicate that increasing the sand proportion reduces the overall strength compared with the 1:1 mix, this is attributed to the fact that increasing the sand content dilutes the cohesive component of the mix which leads to less bonding and an overall reduced strength. The optimum conditions for each plastic level with regard to the DCS were 4.44 MPa at 5% plastic (10% water, 15% gravel), 7.01 MPa at 10% plastic (12.5% water, 5% gravel), and 7.014 MPa at 15% plastic (12.5% water, 25% gravel). Overall, the 1:1 soil-to-sand ratio demonstrated superior compressive performance, highlighting the importance of achieving a balanced granular structure and adequate binder distribution to optimize strength in thermoplastic-stabilized mixtures.

The dry compressive strength results obtained in this study were compared with requirements reported in established international standards for CEB. According to Paul et al.^38^, the minimum compressive strength requirements specified in most international standards range between 1.3 and 5 MPa, depending on the intended structural application. Among these, the British Standard specifies one of the highest minimum requirements, at 5 MPa for dry compressive strength. The optimum mixture developed in this study achieved a dry compressive strength of 9.93 MPa, which significantly exceeds this requirement. This indicates that the proposed blocks meet and surpass the compressive strength criteria typically specified for stabilized earth blocks, demonstrating their potential suitability for masonry applications.

Statistical analysis was performed to evaluate the influence of the soil-to-sand ratio on dry compressive strength. Table 7**.** presents the statistical summary of DCS grouped by soil-to-sand ratio. For the specimens with an S/S ratio of 1:1, the average DCS was 6.21 MPa with a standard deviation of 1.98 MPa and a 95% confidence interval ranging from 5.42 to 6.99 MPa (n = 27). In comparison, specimens with an S/S ratio of 1:1.25 exhibited a lower average DCS of 4.57 MPa, with a standard deviation of 1.70 MPa and a 95% confidence interval between 3.90 and 5.25 MPa (n = 27). An independent t-test indicated that the difference between the two groups is statistically significant (p < 0.05), suggesting that increasing the sand proportion reduces the compressive strength of thermoplastic-stabilized CEBs.

**Table 7:** Statistical summary of DCS for thermoplastic-stabilized CEB specimens grouped by soil-to-sand ratio (S/S).

S/S ratio	Number of specimens (n)	Mean DCS (MPa)	Standard deviation (MPa)	Minimum (MPa)	Maximum (MPa)	95% confidence interval (LCI-UCI) (MPa)
1:1	27	6.21	1.98	3.01	9.93	5.42–6.99
1:1.25	27	4.57	1.70	1.35	7.01	3.90–5.25

To evaluate the relationship between mix design parameters and DCS, both Pearson and Spearman correlation analyses were performed. The correlation coefficients and significance levels are presented in Table 8**.** The results indicate that plastic content exhibits the strongest positive correlation with DCS (Pearson r = 0.451, p = 0.0006; Spearman ρ = 0.506, p = 0.0001), demonstrating a statistically significant relationship and suggesting that increasing thermoplastic content enhances particle bonding and improves the structural integrity of the stabilized compressed earth blocks (CEBs). The soil-to-sand ratio also shows a statistically significant positive correlation with DCS (Pearson r = 0.411, p = 0.002; Spearman ρ = 0.373, p = 0.0055), indicating that mixtures with higher sand proportions tend to exhibit lower dry compressive strength. Conversely, water content (Pearson r =  − 0.003, p = 0.982; Spearman ρ = 0.053, p = 0.7029) and gravel content (Pearson r =  − 0.041, p = 0.771; Spearman ρ =  − 0.041, p = 0.7659) show no statistically significant correlation with DCS within the investigated ranges (p > 0.05). These findings highlight the dominant influence of plastic content on the mechanical performance of the thermoplastic-stabilized CEB mixtures. Overall, the Pearson and Spearman coefficients are in close agreement across all four parameters, confirming the robustness of the observed correlations regardless of the method used.

**Table 8:** Pearson and Spearman correlation coefficients, p-values, and significance for the relationship between mix design parameters and DCS of thermoplastic-stabilized CEB specimens.

Factor	Pearson (r)	p -value	Spearman (ρ)	p-value	Significance
Soil–sand ratio	0.411	0.002	0.373	0.0055	Significant
Plastic content	0.451	0.0006	0.506	0.0001	Significant
Water content	− 0.003	0.982	0.053	0.7029	Not significant
Gravel content	− 0.041	0.771	-0.041	0.7659	Not significant

### Wet compressive strength

**Fig. 7:**
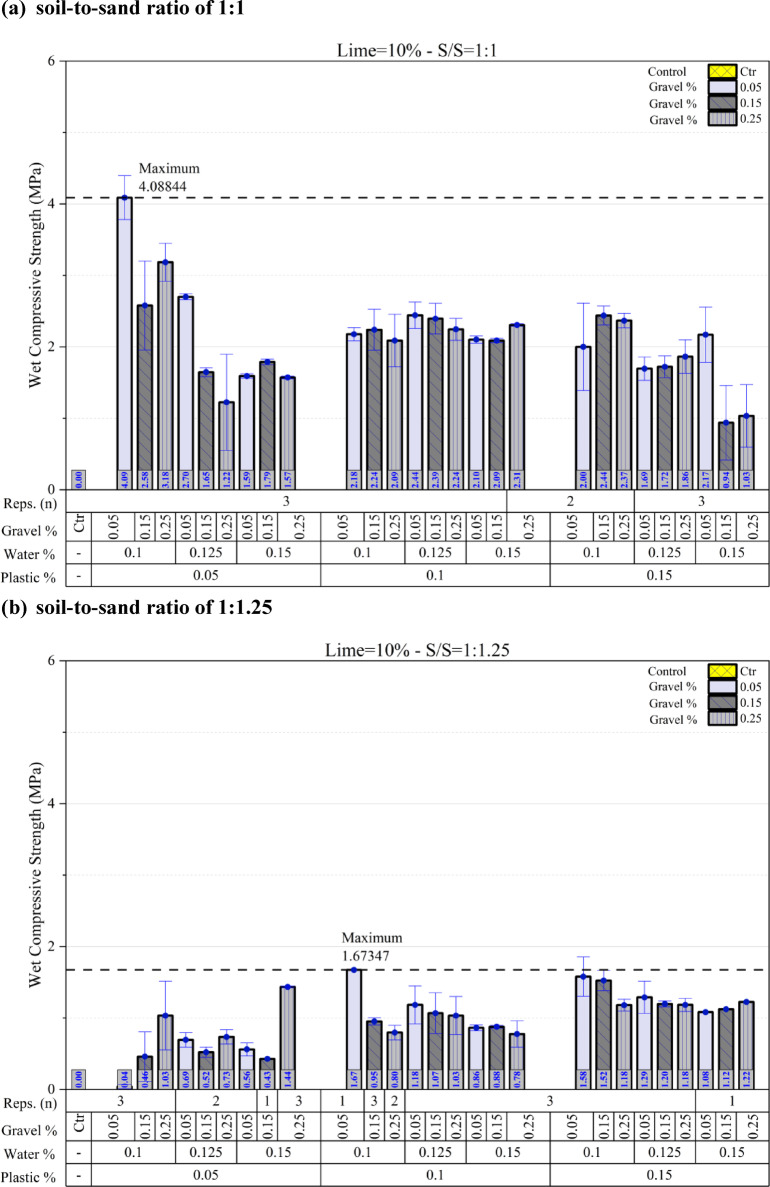
Wet Compressive strength of thermoplastic-stabilized specimens: (**a**) soil-to-sand ratio of 1:1, (**b**) soil-to-sand ratio of 1:1.25. (*Note*: Error bars show the average upper and lower deviation from the mean. Zero-width bars indicate cases where only one specimen succeeded)

Figure 7a and b illustrate the variation in WCS of thermoplastic-stabilized CEB specimens with a fixed lime content of 10% at two different soil-to-sand ratios: 1:1 and 1:1.25, respectively, after 24 h of water immersion. Each bar represents the mean WCS of three replicate specimens (n = 3); for some mixes, specimens were lost to failure during water immersion, reducing the available replicates to two (n = 2) or one (n = 1), as indicated in the replicate row (Reps.). Single-specimen values (n = 1) are shown without error bars, as variability cannot be quantified from one measurement. The unstabilized control specimen exhibited zero WCS, confirming its complete loss of integrity upon saturation and highlighting the critical role of stabilization in providing water resistance. For the 1:1 soil-to-sand ratio (Fig. 7a), the maximum WCS of 4.08 MPa was obtained at 5% gravel, 10% water, and 5% plastic, representing the optimum mix that provides the highest resistance to water exposure. The optimum conditions for each plastic level were 4.08 MPa at 5% plastic (10% water, 5% gravel), 2.44 MPa at 10% plastic (12.5% water, 5% gravel), and 2.44 MPa at 15% plastic (10% water, 15% gravel). For the 1:1.25 soil-to-sand ratio (Fig. 7b), the maximum WCS recorded was 1.67 MPa at 5% gravel, 10% water, and 10% plastic. The overall strength decreased with increasing sand content compared with the 1:1 mix, indicating that excessive sand reduces cohesion and water resistance. The optimum conditions for each plastic level were 1.44 MPa at 5% plastic (15% water, 25% gravel), 1.67 MPa at 10% plastic (10% water, 5% gravel), and 1.58 MPa at 15% plastic (10% water, 5% gravel). Overall, specimens with a 1:1 soil-to-sand ratio exhibited considerably higher WCS than those with a 1:1.25 ratio, confirming that a balanced soil and sand proportion enhances cohesion and water resistance. However, it is noteworthy that the optimum mix for WCS did not coincide with that of the DCS as the optimal mixes for DCS and WCS included 10% and 5% plastic, respectively. This can be attributed to the fact that adding more PP in wet conditions can reduce overall strength due to poor fiber-matrix interaction. Moreover, with less PP, the mix compacts better under wet conditions, resulting in higher WCS. Such discrepancy between the optimum mixes indicates that the parameters enhancing strength under dry conditions differ from those governing performance under water immersion. This distinction highlights the need to balance mix design variables based on the intended environmental exposure of thermoplastic-stabilized CEBs.

Table 9 presents the statistical summary of WCS grouped by soil-to-sand ratio. For the specimens with S/S ratio of 1:1, the average WCS was 2.10 MPa, with a standard deviation of 0.64 MPa and a 95% confidence interval ranging from 1.85 to 2.35 MPa (n = 27). The measured values ranged from 0.94 to 4.09 MPa. In comparison, specimens with an S/S ratio of 1:1.25 exhibited a considerably lower average WCS of 0.98 MPa, with a standard deviation of 0.38 MPa and a 95% confidence interval between 0.83 and 1.13 MPa (n = 27). The corresponding strength values ranged from 0.04 to 1.67 MPa. An independent t-test indicated that the difference between the two groups is statistically significant (p < 0.001), confirming that increasing the sand proportion significantly reduces the wet compressive strength of thermoplastic-stabilized compressed earth blocks (CEBs).

**Table 9:** Statistical summary of WCS for thermoplastic-stabilized CEB specimens grouped by soil-to-sand ratio (S/S).

S/S ratio	Number of specimens (n)	Mean WCS (MPa)	Standard deviation (MPa)	Minimum (MPa)	Maximum (MPa)	95% confidence interval (LCI-UCI) (MPa)
1:1	27	2.10	0.64	0.94	4.09	1.85–2.35
1:1.25	27	0.98	0.38	0.04	1.67	0.83–1.13

To further investigate the influence of mixture parameters on WCS, both Pearson and Spearman correlation analyses were conducted using the complete dataset, with Spearman’s coefficient reported alongside Pearson’s given the ordinal nature of the mix design variables. The correlation coefficients and significance levels are presented in Table 10**.** The soil–sand ratio shows a strong positive correlation with WCS (Pearson r = 0.734, p < 0.001; Spearman ρ = 0.772, p < 0.001), indicating that mixtures with higher sand proportions tend to exhibit lower compressive strength under wet conditions. Plastic content, by contrast, shows only a weak and non-significant association with WCS (Pearson r = 0.039, p = 0.778; Spearman ρ = 0.126, p = 0.3644). Water content (Pearson r =  − 0.251, p = 0.067; Spearman ρ =  − 0.226, p = 0.0999) and gravel content (Pearson r =  − 0.079, p = 0.571; Spearman ρ =  − 0.076, p = 0.5865) likewise show no statistically significant relationship with WCS (p > 0.05). The two methods yield consistent estimates across all parameters, reinforcing the reliability of the soil-to-sand ratio as the principal factor governing wet compressive strength, while the remaining mix design variables show limited association within the tested proportions.

**Table 10:** Pearson and Spearman correlation coefficients, p-values, and significance for the relationship between mix design parameters and WCS of thermoplastic-stabilized CEB specimens.

Factor	Pearson (r)	p -value	Spearman (ρ)	p-value	Significance
Soil–sand ratio	0.734	< 0.001	0.772	< 0.001	Significant
Plastic content	0.039	0.778	0.126	0.3644	Not significant
Water content	− 0.251	0.067	-0.226	0.0999	Not significant
Gravel content	− 0.079	0.571	-0.076	0.5865	Not significant

### Thermal conductivity

**Fig. 8:**
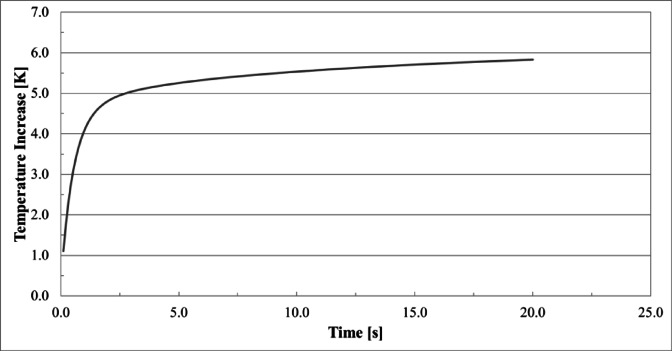
Transient thermal response chart for representative specimen (Specimen 25).

Figure 8 presents a representative transient thermal response curve for one tested pair (Specimen 25) showing the temperature rise over time measured by the TPS apparatus. The curve exhibits a rapid initial increase followed by gradual stabilization, typical of heat diffusion in low-conductivity materials. Similar transient responses were observed for the remaining 54 specimens, with nearly identical behaviour; therefore, only one representative curve is presented to avoid repetition.

The transient temperature response was processed by the Hot Disk analysis software, which fits the recorded ΔT̄(τ) data to the theoretical model defined in Eqs. (2) and (3). Through this fitting procedure, the software simultaneously determines the thermal conductivity (K) and thermal diffusivity (κ) of the material. For Specimen 25, the analysis yielded the thermal properties, with a thermal conductivity of K = 0.834 W·m⁻^1^·K⁻^1^, a thermal diffusivity of κ = 1.77 mm^2^·s⁻^1^, and a volumetric specific heat capacity of 0.471 MJ·m⁻^3^·K⁻^1^. The mean temperature increase recorded at the sensor was 0.489 K, and the total temperature increase over the measurement period reached 5.809 K, confirming that the measurement remained within the valid thermal range specified by ISO 22007–2.

In compliance with ISO 22,007–2 protocols for TPS measurements, thermal conductivity, diffusivity, and volumetric heat capacity of all tested specimens were determined exclusively from the heating transient. The cooling phase, while physically present after the cessation of the 20-s heating pulse, was not analyzed in this study, as the Hot Disk analysis software determines K and κ solely from the temperature rise recorded during the heating pulse, consistent with the method’s theoretical framework established by Gustafsson^46^. The validity of the reported thermal properties is therefore unaffected by this exclusion, as confirmed by the excellent agreement between the measured transient response and the theoretical model, evidenced by mean deviations below 5 × 10⁻^3^ K across all tested specimens.

**Fig. 9:**
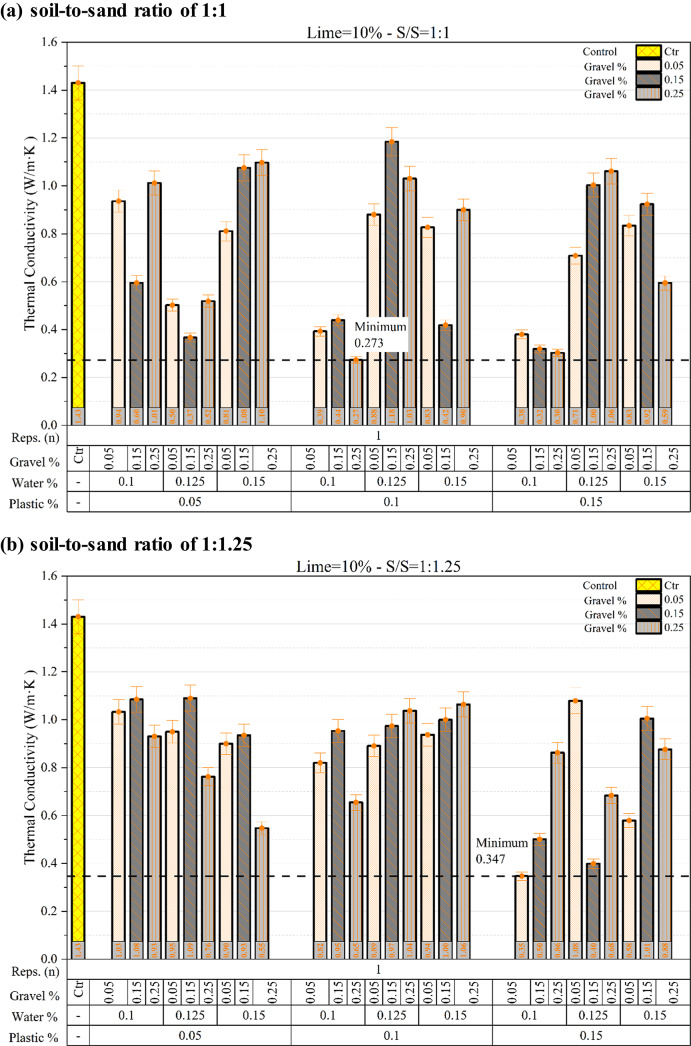
Thermal conductivity of thermoplastic-stabilized specimens: (**a**) soil-to-sand ratio of 1:1, (**b**) soil-to-sand ratio of 1:1.25. (*Note*: Error bars represent a ± 5% uncertainty based on the equipment’s rated accuracy)

Figure 9a and b illustrate the variation in thermal conductivity of thermoplastic-stabilized specimens with a fixed lime dosage of 10% at two different soil-to-sand ratios: 1:1 and 1:1.25, respectively. Each value corresponds to a single thermal conductivity measurement per mix (n = 1), obtained using two specimens of the same mixture in the configuration required by the measurement method. As the test is non-destructive, repeated measurements were not required; the error bars therefore do not represent specimen-to-specimen variability but rather a ± 5% uncertainty based on the equipment’s rated accuracy. The results confirm that thermal conductivity is highly sensitive to the combined influence of gravel, water, and plastic contents, exhibiting a nonlinear relationship that reflects complex interactions between particle arrangement, moisture distribution, and thermoplastic bonding. The unstabilized control specimen recorded a thermal conductivity of 1.43 W/m·K, the highest value among all mixes, indicating the poorest insulation performance and demonstrating the substantial improvement achieved through thermoplastic stabilization. For the 1:1 soil-to-sand ratio (Fig. 9a), the lowest thermal conductivity of 0.27 W/m·K was achieved at 10% plastic, 25% gravel, and 10% water, representing the optimum mix for enhanced insulation. This composition provided the best balance between aggregate packing and polymer dispersion, resulting in reduced heat transfer through the matrix. The optimum values for each plastic level were 0.37 W/m·K at 5% plastic (15% gravel, 12.5% water), 0.27 W/m·K at 10% plastic (25% gravel, 10% water), and 0.30 W/m·K at 15% plastic (25% gravel, 10% water). For the 1:1.25 soil-to-sand ratio (Fig. 9b), a minimum thermal conductivity of 0.35 W/m·K was recorded at 15% plastic, 5% gravel, and 10% water, indicating the best insulation performance for this mix. The optimum values for each plastic level were 0.55 W/m·K at 5% plastic (25% gravel, 15% water), 0.65 W/m·K at 10% plastic (25% gravel, 10% water), and 0.35 W/m·K at 15% plastic (5% gravel, 15% water).

A comparison between both ratios shows that increasing sand content (from 1:1 to 1:1.25) shifts the optimum mix toward higher plastic and lower gravel, leading to higher thermal conductivity and weaker insulation. The 1:1 mix demonstrated superior thermal resistance, indicating that lower sand content and a more balanced granular composition enhance heat insulation. It is noteworthy that as the sand content increases in a soil mix, thermal conductivity also increases because sand particles conduct heat more effectively than clay or other soil particles, and they reduce air gaps within the mix, leading to higher overall conductivity. The observed non-linear response reflects the complex interaction between thermal pathways, particle contacts, and polymer distribution in governing conductivity.

Table 11 presents the statistical summary of thermal conductivity grouped by soil-to-sand ratio. For specimens with a soil-to-sand (S/S) ratio of 1:1, the average thermal conductivity was 0.72 W/m·K, with a standard deviation of 0.29 W/m·K and a 95% confidence interval of 0.60–0.83 W/m·K (n = 27). Individual measurements ranged from 0.27 to 1.18 W/m·K. Specimens with an S/S ratio of 1:1.25 showed a higher mean thermal conductivity of 0.85 W/m·K, with a standard deviation of 0.22 W/m·K and a 95% confidence interval of 0.76–0.93 W/m·K (n = 27). Their thermal conductivity values ranged from 0.35 to 1.09 W/m·K. An independent t-test indicated that the difference between the two groups was not statistically significant (p > 0.05), although the 1:1 ratio exhibited slightly lower thermal conductivity, indicating better insulation performance on average.

**Table 11:** Statistical summary of thermal conductivity for thermoplastic-stabilized CEB specimens grouped by soil-to-sand ratio (S/S).

S/S ratio	Number of specimens (n)	Mean thermal conductivity (W/m·K)	Standard deviation (W/m·K)	Minimum (W/m·K)	Maximum (W/m·K)	95% confidence interval (LCI-UCI) (W/m·K)
1:1	27	0.72	0.29	0.27	1.18	0.60–0.83
1:1.25	27	0.85	0.22	0.35	1.09	0.76–0.93

To assess the influence of mixture parameters on thermal conductivity, both Pearson and Spearman correlation analyses were performed, as presented in Table 12. Lower thermal conductivity corresponds to better insulation performance. Water content showed a statistically significant positive correlation by Pearson (r = 0.305, p = 0.025), indicating that higher water content increases thermal conductivity and reduces insulation performance; however, the Spearman coefficient for water content did not reach significance (ρ = 0.2532, p = 0.0647), and since water content is a continuous variable, the Pearson result is taken as the primary measure. Plastic content showed a weak negative correlation by both methods (Pearson r =  − 0.234, p = 0.088; Spearman ρ =  − 0.2503, p = 0.0679), suggesting a potential, though not statistically confirmed, reduction in thermal conductivity and improvement in insulation with higher plastic content. The soil–sand ratio (Pearson r =  − 0.250, p = 0.068; Spearman ρ =  − 0.2222, p = 0.1064) showed no statistically significant association with thermal conductivity. Gravel content (Pearson r = 0.035, p = 0.801; Spearman ρ = 0.0786, p = 0.5722) likewise showed no significant correlation. These results indicate that water content is the most influential factor affecting thermal conductivity, while the other mixture variables show limited and statistically unconfirmed effects within the tested range.

**Table 12:** Pearson and Spearman correlation coefficients, p-values, and significance for the relationship between mix design parameters and thermal conductivity of thermoplastic-stabilized CEB specimens.

Factor	Pearson (r)	p -value	Spearman (ρ)	p-value	Significance
Soil–sand ratio	− 0.250	0.068	− 0.2222	0.1064	Not significant
Plastic content	− 0.234	0.088	− 0.2503	0.0679	Not significant
Water content	0.305	0.025	0.2532	0.0647	Significant
Gravel content	0.035	0.801	0.0786	0.5722	Not significant

### High performance thermoplastic-stabilized CEB mixes

**Fig. 10:**
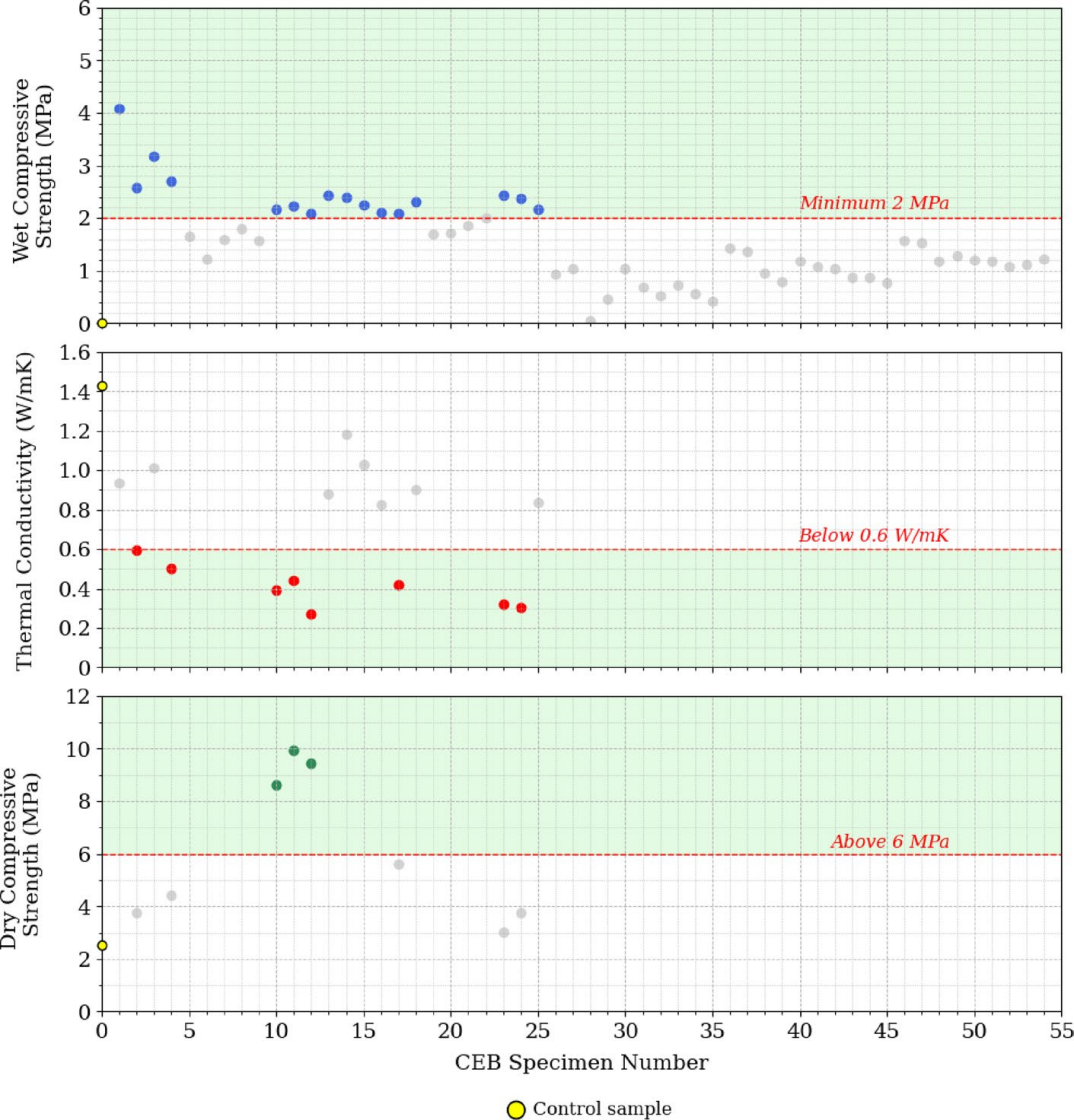
High Performance of thermoplastic-stabilized CEB mix designs.

A selection framework was applied to identify the high-performance mix designs for the thermoplastic-stabilized CEB specimens. The process began with assessing the WCS after 24 h of water immersion for 54 mix designs, to identify those meeting the minimum requirement of 2 MPa for WCS, as specified by the Egyptian Earth Construction Code for non-load-bearing walls up to two stories^47^. Figure 10 shows that only 16 specimens out of 54 (highlighted in blue) satisfied this criterion. The results indicate that all specimens beyond Specimen 28, which had a soil-to-sand ratio of 1:1.25, failed to meet the strength requirement. This suggests that increasing sand content beyond the 1:1 ratio reduces water resistance. Moreover, Specimens 5 to 9, containing 5% plastic and higher water content, also failed, demonstrating that excessive water combined with low plastic content decreases durability under water exposure. Subsequently, the thermal conductivity of the 16 passing specimens was analyzed to identify those offering superior insulation performance. Specimens with conductivity values below 0.6 W/m·K, considered the upper acceptable limit for thermal insulation^48^, were selected, resulting in 8 qualified samples (highlighted in red). While no direct linear correlation was observed, half of these specimens contained 10% plastic, indicating a possible relationship between plastic addition and improved insulation performance. Such a relationship is expected, as adding PP, which is more insulating than soil, reduces the overall ability of the mix to conduct heat. Moreover, PP introduces voids and air gaps into the mix, resulting in less dense material and, consequently, lower thermal conductivity. Finally, the DCS of the eight thermally efficient specimens were reassessed, and those exceeding 6 MPa, which is the upper target range for DCS specified in the Egyptian Code^47^, were highlighted in green to indicate the high-performance mixes.

Three specimens showed consistently high performance across DCS, WCS and thermal conductivity, corresponding to the mix ratios summarized in Table 13. All three exhibited a soil-to-sand ratio of 1:1, with 10% lime, 10% water, and 10% plastic contents. The main variation among them was in the gravel content, which ranged from 5 to 25%. These mixes demonstrated the best overall balance between compressive strength, water resistance, and thermal insulation, making them the most suitable for sustainable non-load-bearing construction. Its key to note that while gravel affects strength and thermal behavior, its influence is less dominant when fines (soil + sand) are optimized, lime stabilization is utilized, and plastic fibers are incorporated to help bind the matrix. Also, even with 5–25% gravel, the dominant influence on insulation performance came from the plastic content and fines, which were constant.

**Table 13:** Mix compositions and corresponding high-performance results for thermoplastic-stabilized CEB specimens.

S/S ratio	Gravel (%)	Lime (%)	Water (%)	Plastic (%)	Dry compressive strength (MPa)	Wet compressive strength (MPa)	Thermal conductivity (W/m·K)
1:1	5	10	10	10	8.63	2.18	0.39
1:1	15	10	10	10	9.93	2.24	0.44
1:1	25	10	10	10	9.44	2.09	0.27

### Correlation between testing results

**Fig. 11:**
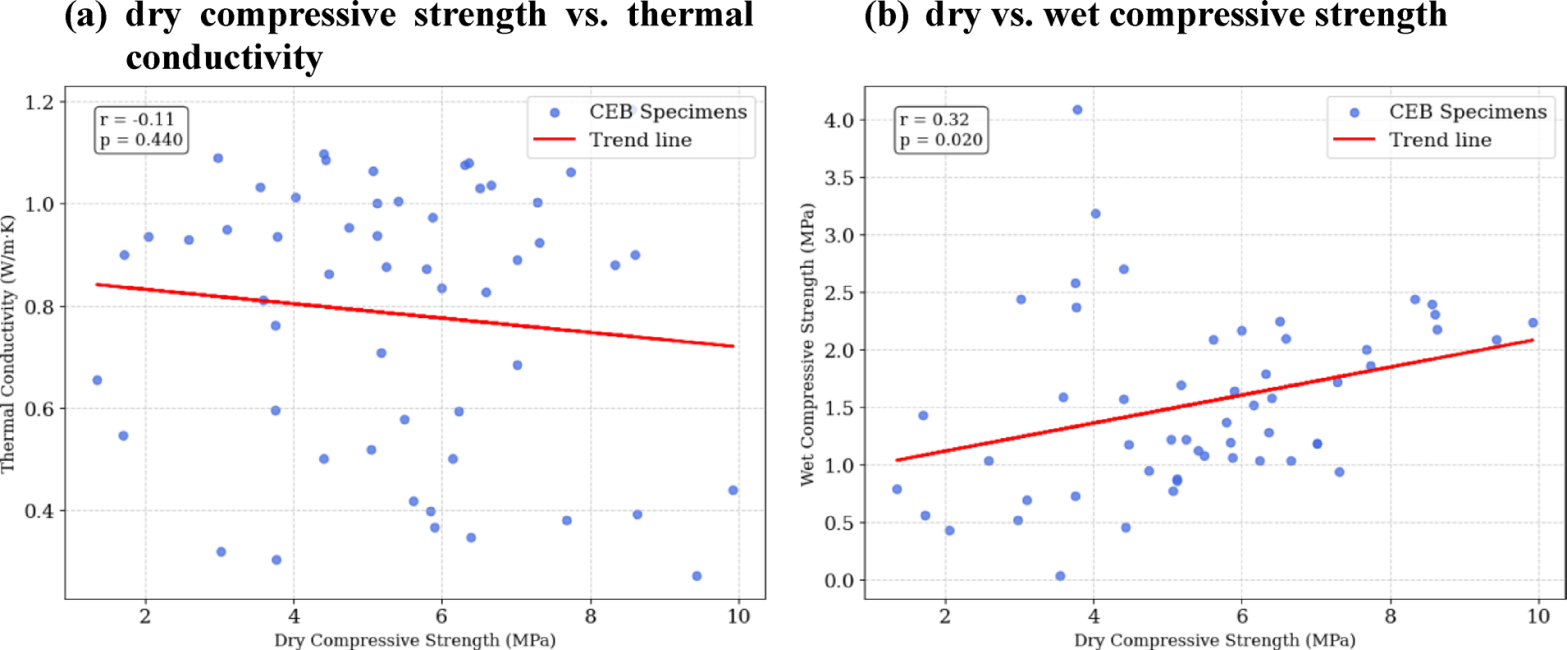
Correlation analysis of thermoplastic-stabilized CEB specimens: (**a**) dry compressive strength vs. thermal conductivity, (**b**) dry vs. wet compressive strength.

A correlation analysis was conducted to examine the relationships among the key measured properties of thermoplastic-stabilized CEBs, namely DCS, WCS, and thermal conductivity. Figure 11a presents the relationship between DCS and thermal conductivity, with a Pearson correlation coefficient of − 0.11 and a corresponding p -value of 0.440. This weak negative correlation indicates a slight tendency for stronger specimens to exhibit lower thermal conductivity; however, the high p -value confirms that the relationship is not statistically significant. Importantly, this result shows that there is no evident trade-off between mechanical strength and thermal insulation. Therefore, both high compressive strength and low thermal conductivity can be achieved concurrently in optimized mixes, allowing for simultaneous improvement of both structural and thermal performance without compromise. Moreover, Fig. 11b illustrates the correlation between DCS and WCS after 24-h water immersion, which produced a Pearson correlation coefficient of 0.32 with a p -value of 0.020. This moderate positive correlation suggests that specimens with higher DCS generally maintained higher strength after immersion, and the statistically significant p -value indicates that this relationship is unlikely to be due to random variation. Such behaviour aligns with expected material response, as denser and stronger matrices typically demonstrate improved durability under water exposure. Although the correlation is not particularly strong, its statistical significance supports the reliability and internal consistency of the experimental data, indicating that the observed variations in strength are systematic rather than random.

The strength improvements recorded in thermoplastic-stabilized CEBs are not attributable to a single dominant process but rather appear to emerge from the interplay of several concurrent mechanisms. When subjected to heat and compaction pressure, PP particles are expected to soften and migrate into the void spaces between soil and sand grains, forming upon cooling a discontinuous yet potentially interconnected polymeric network that may reinforce particle-to-particle contacts and resist crack propagation under load. This binder-induced cohesion is thought to act in conjunction with cementitious products that may be generated through lime-soil pozzolanic reactions, principally C–S–H and C–A–H gels, which are believed to progressively coat particle surfaces and occlude micropores, thereby contributing to matrix stiffening independently of the thermoplastic phase. These interpretations, however, are based on observed macroscopic behavior and established mechanisms reported in the literature, as no direct microstructural characterization was carried out in this study. The granular framework itself is considered to play an equally important role. At moderate gravel contents, a well-interlocked coarse skeleton is thought to be established, into which the molten binder can distribute more uniformly, whereas at higher gravel proportions the inter-grain voids may exceed the capacity of the available binder to fill them, reducing the effective bonded contact area. Water content is understood to govern compaction quality during fabrication, with moderate levels likely facilitating grain rearrangement and intimate binder-particle contact, while excess moisture is expected to introduce discontinuities within the binder phase through vapor generation during pressing. The divergence between optimum dry and wet compressive strength mixes is attributed to the hydrophobic character of PP, which is believed to render the PP-soil interface susceptible to moisture infiltration under saturation; at lower PP contents the matrix appears to be governed more by lime-cementation, whose products are considered inherently less vulnerable to this form of interfacial degradation. Collectively, these proposed mechanisms offer a plausible explanation for both the magnitude of the observed strength gains and the nonlinear dependence of performance on individual mix variables, pending future microstructural validation.

### Economic feasibility assessment

A preliminary cost assessment was conducted to estimate the potential economic feasibility of the proposed blocks. The material prices used in this estimate reflect typical values in the Egyptian market as of June 2025. The assessed costs were 8000 LE per ton for recycled plastic, 1000 LE per ton for lime, and 20 LE per m^3^ for water. It was further assumed that soil, sand, and gravel would be sourced directly on-site, which is common practice in earth-based construction and significantly reduces transportation and raw material costs. Based on the proportions of the optimum mixture, the appraised unit production cost was approximately $33.8 per 1000 bricks. For comparison, conventional concrete bricks are valued at approximately $33 per 1000 bricks. This value only includes raw material costs and does not account for fixed expenses such as equipment, labor, energy consumption, or transportation, which may vary significantly depending on production scale and location. Additionally, the proposed blocks eliminate cement use and incorporate recycled plastic waste, which may offer environmental advantages and potential long-term economic benefits.

### Comparative performance with recent CEB studies

Comparison with the recent CEB stabilization studies presented in Table 1 provides further insight into the relative performance of the proposed thermoplastic-stabilized CEBs. In terms of dry compressive strength, the present blocks (up to 9.93 MPa) outperform natural fiber-stabilized systems, including Acrea fibers (2.28 MPa)^27^ and vetiver straw fibers (1.36 MPa)^28^, as well as the sewage sludge ash–cement system (5.42 MPa)^34^. Their strength is comparable to ceramic-waste-powder (8.96 MPa)^35^ and crushed-brick-waste (9.57 MPa)^36^ systems, but remains below the fly ash–sewage sludge ash geopolymer (11.36 MPa)^31^, recycled-cement (10.9 MPa)^32^, and blast-furnace-slag/cement/lime (14.31 MPa)^33^ systems, all of which incorporate Portland cement or an equivalent hydraulic binder. Importantly, the present compressive strength is achieved without cement, whereas all of the higher-strength comparator systems rely on cement, either alone or in combination with supplementary cementitious materials or industrial by-products.

In terms of durability, the present blocks exhibited a wet compressive strength (WCS) of 4.08 MPa, which exceeds that of the Acrea fiber-stabilized blocks (1.60 MPa)^27^ but remains below the cement-stabilized comparator systems, whose WCS values range from 5.0 MPa for ceramic-waste-powder blocks^35^ to 9.43 MPa for the fly ash–sewage sludge ash geopolymer system^31^. The vetiver fiber study reported water absorption but not WCS^28^.

For thermal conductivity, the present study reports the only quantified value among the compared entries (as low as 0.27 W/m·K), as none of the six comparator studies report this property. This represents a distinct contribution of the present work, since thermal performance is rarely characterized in the recent CEB stabilization literature despite its direct relevance to operational energy demand.

On cost and sustainability, the present study and the ceramic-waste-powder study^35^ are the only two among the seven to report quantified cost figures, though on different bases: the present study provides an absolute raw-material cost (≈$33.8 per 1000 blocks) benchmarked against conventional concrete bricks, while the ceramic-waste-powder study reports a relative reduction (≈11% cost saving from cement substitution) via life-cycle assessment (LCA). The remaining comparator studies emphasize qualitative sustainability benefits, such as industrial by-product diversion, kiln-firing elimination, without quantifying cost. The present study’s contribution to sustainability is distinguished by its complete elimination of cement, and by its valorization of recycled plastic.

## Conclusion

This research investigated the performance of thermoplastic-stabilized compressed earth blocks (CEBs) produced using shredded waste PP under heat application as a sustainable alternative to conventional materials. The experimental program, comprising 54 mix designs, examined the effects of plastic, water, soil, sand, and gravel contents on the mechanical strength, durability, and thermal insulation of lime-stabilized CEBs.

Most thermoplastic-stabilized mixes exceeded the control dry strength of 2.55 MPa, with several reaching more than three times this value, demonstrating the clear mechanical benefits of thermoplastic addition. The unstabilized control showed zero wet strength, confirming its complete failure under saturation and the need for stabilization to ensure water resistance. It also had the highest thermal conductivity (1.43 W/m·K), indicating the poorest insulation and highlighting the thermal improvements achieved through thermoplastic stabilization.

Results showed that the 1:1 soil-to-sand ratio consistently outperformed the 1:1.25 ratio in both dry and wet compressive strength. The optimum dry strength of 9.93 MPa was achieved at 15% gravel, 10% water, and 10% plastic, while the highest wet strength of 4.08 MPa occurred at 5% gravel, 10% water, and 5% plastic. The lowest thermal conductivity of 0.27 W/m·K was recorded at 10% plastic, 25% gravel, and 10% water, highlighting improved insulation with higher plastic content.

Correlation analysis provided further insight into the influence of mixture parameters on CEB performance. Plastic content showed the strongest positive correlation with dry compressive strength (Pearson r = 0.451, p = 0.0006; Spearman ρ = 0.506, p = 0.0001), confirming its major role in enhancing particle bonding and matrix densification. The soil–sand ratio also demonstrated a moderate positive correlation with DCS (Pearson r = 0.411, p = 0.002; Spearman ρ = 0.373, p = 0.0055), indicating that higher sand proportions tend to reduce strength. In contrast, water content and gravel content showed no statistically significant influence on dry strength. For wet compressive strength, the soil–sand ratio emerged as the dominant parameter (Pearson r = 0.734, p < 0.001; Spearman ρ = 0.772, p < 0.001), whereas plastic, water, and gravel contents exhibited weak, statistically non-significant effects. Thermal conductivity was most influenced by water content, which displayed a significant positive Pearson correlation (r = 0.305, p = 0.025), indicating reduced insulation at higher water contents; Pearson correlation was used as the primary measure because water content is a continuous variable. Plastic content showed a weak negative, non-significant trend (Pearson r =  − 0.234, p = 0.088; Spearman ρ =  − 0.2503, p = 0.0679), suggesting a potential but unconfirmed improvement in insulation performance.

The selection framework identified three high-performance mix combinations., all with 10% lime, 10% water, 10% plastic, and a 1:1 soil-to-sand ratio, differing only in gravel content (5%, 15%, and 25%). These mixes demonstrated a well-balanced performance across strength, improved durability, and insulation criteria. The mix with 5% gravel achieved a dry compressive strength of 8.63 MPa, wet strength of 2.18 MPa, and thermal conductivity of 0.39 W/m·K. The 15% gravel mix yielded the highest dry strength of 9.93 MPa, wet strength of 2.24 MPa, and thermal conductivity of 0.44 W/m·K. Meanwhile, the 25% gravel mix exhibited a dry strength of 9.44 MPa, wet strength of 2.09 MPa, and the lowest thermal conductivity of 0.27 W/m·K, indicating superior insulation capacity.

A weak negative relationship between dry compressive strength and thermal conductivity (r =  − 0.11), indicating that higher strength can be achieved without sacrificing insulation. A moderate positive correlation between dry and wet compressive strengths (r = 0.32) further showed that stronger specimens maintained better durability under water exposure, confirming the role of matrix density and thermoplastic bonding in enhancing performance.

A preliminary economic assessment showed that the proposed thermoplastic-stabilized blocks are cost-competitive, with an estimated production cost of $33.8 per 1000 bricks, closely matching the price of conventional concrete bricks (~ $33 per 1000 units). This estimate is limited to raw material costs and does not represent a full life-cycle cost evaluation.

Benchmarking indicates that the proposed thermoplastic-stabilized CEBs achieve dry compressive strength competitive with alternative additives studies, while showing lower wet compressive strength than most benchmarked systems with durability data, a trade-off attributable to the absence of cement. The present blocks offer a unique thermal-performance dataset absent from the compared literature, and a distinct sustainability proposition centered on complete cement elimination and plastic-waste valorization, as opposed to the partial cement reduction achieved via industrial by-product substitution in the benchmarked studies.

While the use of recycled plastic and the elimination of cement may indicate potential environmental advantages, these aspects were not quantified through LCA or carbon accounting in this study. Therefore, sustainability-related conclusions are limited to qualitative observations.

## Data Availability

The datasets generated during and/or analyzed during the current study are available from the corresponding author on reasonable request.
